# Pandemic health consequences: Grasping the long COVID tail

**DOI:** 10.1371/journal.pmed.1003891

**Published:** 2022-01-25

**Authors:** Kieran L. Quinn, Chaim M. Bell

**Affiliations:** 1 Department of Medicine, University of Toronto, Toronto, Canada; 2 ICES, Toronto, Canada; 3 Department of Medicine, Sinai Health System, Toronto, Canada; 4 Temmy Latner Centre for Palliative Care, Sinai Health System, Toronto, Canada

Emerging evidence suggests that approximately 10% of people who survive Coronavirus Disease 2019 (COVID-19) will have lingering symptoms that negatively affect their quality of life, ability to work, and function [1,2]. This important group of people with the post-COVID-19 condition may seem small in comparison to the overall number of people with COVID-19 infection [3]. However, many patients who survive COVID-19 are likely to have considerable symptom burden, high resource utilization and health service needs, reduced economic productivity, and possibly a shortened life expectancy. The study by Bhaskaran and colleagues published in *PLOS Medicine* addresses an evolving, poorly studied, and important area of health policy and planning related to the care of patients who survive hospitalization for COVID-19 [4].

At face value, the scope of the COVID-19 pandemic is enormous. Within 2 years, nearly 300 million people have been infected with the Severe Acute Respiratory Syndrome Coronavirus 2 (SARS-CoV-2) virus, and more than 5 million people have died from it [5]. But, there is also a long tail to this statistical distribution of hardship. Studies report that numerous patients will continue to experience fatigue, shortness of breath, pain, sleep disturbances, anxiety, and depression [6]. More serious organ dysfunction such as pulmonary fibrosis, cognitive impairment, myocarditis, and renal failure may also develop [6]. Whether these translate into clinical diagnoses of chronic diseases like interstitial lung disease, dementia, heart failure, and chronic kidney disease remains to be seen. Collectively, the prospect for immense suffering among these individuals will undoubtedly have huge and enduring impacts on healthcare systems globally. As the world continues its largest vaccination effort in history and looks to eliminate the impacts of acute COVID-19, we must not forget that a meaningful minority who survive will transition from an acute to chronic disease state. In turn, management strategies and health resource planning must also appropriately transition. As a multisystem disease, the post-COVID-19 condition will require the involvement of multidisciplinary care teams [7]: Who will help to look after these patients?

Bhaskaran and colleagues studied over 164,000 hospitalized adults with COVID-19 matched to an “active control” group of adults hospitalized with influenza and to general population controls. They compared the medium- and long-term risks of hospital admission and death across the 3 study groups. The main findings were that people discharged following hospitalization for COVID-19 had a 2-fold higher associated risk for rehospitalization and death than the general population and similar risks compared to those hospitalized for influenza. These outcomes were most pronounced in the first 30 days following discharge yet remained substantially elevated over time. Further, those hospitalized with COVID-19 were more likely to be rehospitalized or die from mental health or cognitive-related causes, especially if they had preexisting dementia, compared to those hospitalized with influenza.

Initial hospitalization with COVID-19 represents a crucial touch point within the healthcare system. The study by Bhaskaran and colleagues sheds important light on the health service needs of patients who survive hospitalization for COVID-19. It further helps disentangle the effects of hospitalization from respiratory viral infection on important outcomes. The current work builds on similar findings from a recent study of 47,780 hospitalized adults with COVID-19 who survived to discharge with a mean follow-up of 140 days [8]. In that study, rates of hospital readmission and mortality were 3.5 and 7.7 times greater in the previously hospitalized group of COVID-19 patients, compared to general population controls, respectively. Other studies from the United States and China followed patients hospitalized for COVID-19 and reported lower 60-day and 1-year rehospitalization rates ranging from 13% to 19.9%. However, these studies did not account for the competing risk of death as was done in the current study [9–11].

There were also noteworthy limitations of Bhaskaran and colleagues’ study. First, cause-specific outcomes among adults with COVID-19 may be artificially higher than those with influenza due to availability bias. Put simply, patients and providers may be much more aware of COVID-19 and its complications, including those related to return to hospital, than might be the case for those with pneumonia or even confirmed influenza. Second, the study used administrative data from primary care. While 98% of the population in England are registered with a general practice (thereby minimizing selection biases due to health-seeking behaviors), there are some geographical differences in the use of the OpenSAFELY platform, which may introduce the potential for selection bias. Third, this study was conducted in a high-income nation with substantial resources to support patients following infection with COVID-19. The generalizability of these findings to middle- and low-income nations, or those with limited resources, is unknown.

The study by Bhaskaran and colleagues has clear applications to healthcare resource planning and policy in the care of individuals who survive COVID-19. This suggests a substantial added burden on global healthcare systems. It further builds on our evolving knowledge of the post-COVID-19 condition and its lingering impacts, including on many previously healthy adults in their prime years of productivity. Still, a wealth of research is required to develop prediction tools to proactively identify and support the healthcare needs of survivors, including end-of-life care, develop new strategies to prevent and treat the post-COVID-19 condition, and encourage interprofessional teams to provide longitudinal care through innovative health policy interventions.

Early pandemic public messaging strategies focused on flattening the peak of the acute COVID-19 infection curve to preserve healthcare system capacity and its ability to deliver high-quality care. These efforts were generally successful. To preserve ongoing system capacity and provide high-quality patient care, the long COVID tail does not require further flattening, but rather demands new clinical and health policy strategies to address its potential for long-term suffering. Here, we must recognize that the head of the pandemic often demands our immediate attention, but we must not ignore its long and deadly tail.
